# Combined Oral Contraceptive Drug–Drug Interaction Study With Ganfeborole, a New Anti‐Tuberculosis Agent

**DOI:** 10.1002/jcph.70161

**Published:** 2026-03-06

**Authors:** Laura Iavarone, Silvia M. Lavezzi, Antonio J. Carcas, Tetyana Chaychenko, Raquel Gabarro‐Carrion, Arturo Gómez López de las Huertas, Stephanie Gresham, Alicia Marín‐Candón, Sophie L. Penman, Katie Rolfe, Simon Tiberi, David Barros‐Aguirre, Alberto M. Borobia, the ERA4TB consortium

**Affiliations:** ^1^ Parexel International Milan Italy; ^2^ Parexel International Dublin Ireland; ^3^ Clinical Pharmacology Department La Paz University Hospital IdiPAZ, and School of Medicine Universidad Autónoma de Madrid Madrid Spain; ^4^ GSK London UK; ^5^ GSK Tres Cantos Spain; ^6^ GSK Stevenage UK; ^7^ Blizard Institute Barts and The London School of Medicine and Dentistry Queen Mary University of London London UK

**Keywords:** drug–drug interaction, ethinyl estradiol, ganfeborole, levonorgestrel, pharmacokinetics, tuberculosis

## Abstract

New drugs are urgently needed to treat drug‐resistant tuberculosis in combination regimens. Ganfeborole demonstrated bactericidal activity and good tolerability in clinical trials. In preclinical studies, ganfeborole showed embryofetal developmental effects, currently mandating highly effective non‐user dependent contraception in women of childbearing potential. We conducted a Phase 1, open‐label, single‐center, fixed sequence, 1‐way drug–drug interaction (DDI) study in 20 healthy women of non‐childbearing potential aged 18‐65 years. The primary objective was to assess ganfeborole's effect at steady‐state (20 mg daily) on single dose pharmacokinetics of ethinyl estradiol [EE] 0.03 mg/levonorgestrel [LNG] 15 mg (Bayer). Endpoints were EE and LNG area under the plasma concentration‐time curve extrapolated to infinity (AUC_(0‐inf)_) and maximum concentration (*C*_max_). Unexpected fluctuations in individual EE and LNG plasma concentration‐time profiles limited the number of acceptable endpoints for the analysis. Geometric mean ratios (GMR; EE/LNG+ganfeborole versus EE/LNG alone) and respective 90% confidence intervals (CI) for EE *C*_max_ (0.96, 0.85‐1.09), LNG AUC_(0‐inf)_ (1.10, 0.98‐1.23) and LNG *C*_max_ (1.08, 0.97‐1.19) met criteria for lack of DDI (90% CI 0.80‐1.25). However, the GMR for EE AUC_(0‐inf)_ was 0.88, with 90% CI 0.55‐1.41. While post‐hoc analyses on partial AUCs (up to 8 and 24 h) provided GMR 90% CIs within 0.80‐1.25, a lack of DDI could not be concluded. No treatment‐related adverse events were reported. Further assessments of potential DDI between ganfeborole and combined oral contraceptives are warranted. Future trials will maintain strict contraception requirements.

Clinical Trial Registration: NCT06354257 (registration date: 2024‐04‐03); EudraCT: 2023‐507839‐38‐00

## Introduction

Tuberculosis (TB) is caused by the bacterium *Mycobacterium tuberculosis*.^1^ The number of people who developed TB in 2023 was estimated as 10.8 million, and it is the leading cause of death resulting from an infectious disease, with approximately 1.25 million attributable fatalities in 2023.^1^ Approximately 20% of new and relapsed TB cases reported each year are in women 15‐44 years of age, i.e. of childbearing potential, and TB poses risks in pregnancy to both the mother and the developing fetus.^1^, ^2^, ^3^

The standard of care for drug‐susceptible TB is a combination of rifampicin, isoniazid, pyrazinamide, and ethambutol.^4^ However, drug‐resistant TB is a major public health problem.^1^, ^5^ While treatment regimens for multi‐drug resistant TB have become less complex and of shorter duration, toxicity and access remain challenges.^6^ Although several new drugs have emerged recently, resistance to those has also been reported.^4^ In particular, bedaquiline resistance is a growing threat that is of great concern.^7^ There is a clear need for new drugs that can be used as part of a combination regimen.^4^

Ganfeborole (GSK3036656) is a new 3‐aminomethyl 4‐chlorobenzoxaborole which selectively inhibits the *M. tuberculosis* leucyl‐tRNA synthetase.^8^, ^9^ Pharmacokinetic (PK) analysis showed a dose‐proportional increase in concentration with single‐dose administration of ganfeborole 5‐25 mg and repeat‐dose administration of ganfeborole 5 and 15 mg daily over 14 days in healthy volunteers.^10^ Unchanged ganfeborole was the only circulating drug‐related component detected after both single and repeat dosing.^10^ Urinary excretion accounted for between 50% and 78% of the dose (up to 72 h post‐dose) following single and repeat administration, 90% of which was unchanged ganfeborole.^10^ Based on in vitro data, ganfeborole is not expected to perpetrate drug–drug interactions at the cytochrome P450 level. However, ganfeborole has shown embryofetal development effects in rats and rabbits; in rats (the most sensitive species), such effects were observed at doses equivalent to clinical therapeutic exposure [data on file; GSK document No. RPS‐CLIN‐162271].

Ganfeborole has now entered Phase 2 clinical trials^11^ and has shown promising bactericidal activity and was well tolerated as monotherapy in a Phase 2a clinical trial (NCT03557281).^12^ A Phase 2a early bactericidal activity study of ganfeborole as part of a 2‐drug combination with established and newer anti‐TB agents under the CLICK‐TB consortium (NCT05382312) and a Phase 2b/c regimen‐building and duration‐ranging study conducted under the UNITE4TB (Academia and Industry United Innovation and Treatment for Tuberculosis) consortium (NCT06114628) started in 2024.^13^, ^14^

To minimize any risk following the embryofoetal developmental effects observed in rats and rabbits, clinical development studies including ganfeborole currently mandate the use of highly effective non‐user dependent contraception (intrauterine contraceptive device or implantable or depot injectable progesterone) for female participants of childbearing potential, or recruitment of women of non‐childbearing potential. In addition, pregnancy testing (serum or high sensitivity urine tests) must be performed prior to dosing and throughout the studies.

As non‐user dependent contraceptive measures may not be available or culturally acceptable in some countries, providing the opportunity to use user‐dependent oral contraception would allow more women to participate in ganfeborole treatment development programmes by offering greater choice. Despite no known mechanistic basis for interaction between ganfeborole and oral contraceptives, to comply with regulatory recommendations and address the teratogenicity concern,^15^, ^16^ we have conducted a drug–drug interaction study to evaluate the impact of ganfeborole on the pharmacokinetics of the components of a commonly available combined oral contraceptive (ethinyl estradiol [EE] and levonorgestrel [LNG]), and to inform inclusion/exclusion criteria for the next drug development phases. The study has been supported by the European Regimen Accelerator for Tuberculosis (ERA4TB) consortium, a public‐private initiative of more than thirty organizations from the European Union and the United States whose primary aim is to accelerate the development of new TB treatment regimens.^17^ ERA4TB is part of the antimicrobial resistance (AMR) accelerator, which is an Innovative Medicines Initiative programme involving nine projects aimed at developing new drugs for resistant bacterial infections.^18^

**Figure 1:**
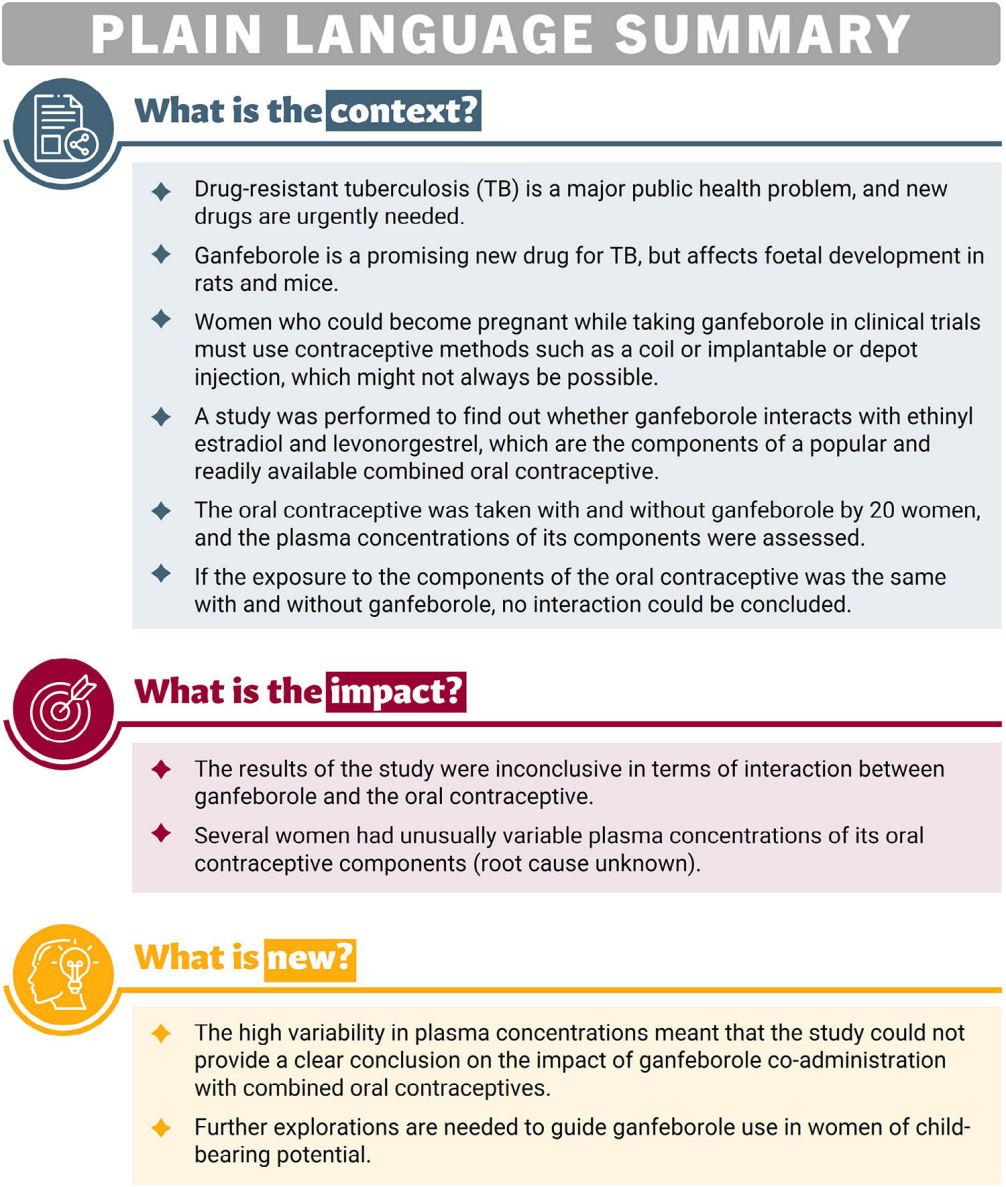
Plain Language Summary.

Figure 1 provides a plain language summary of the drug–drug interaction study.

## Methods

The protocol was approved by the independent ethics committee and institutional review board of the Hospital La Paz, Madrid, Spain where the study took place. Participants provided written informed consent at enrolment. The study was conducted in accordance with the Declaration of Helsinki guidelines and the principles of Good Clinical Practice.

The primary objective of the study was to assess the effect of ganfeborole steady‐state exposure on the PK of a single dose of the combined oral contraceptive, EE 0.03 mg/LNG 15 mg (Bayer), in healthy women of non‐childbearing potential.

### Study Design, Participants, and Drugs

This was a Phase 1, open‐label, single‐center, fixed sequence, 1‐way drug–drug interaction study (NCT06354257; EudraCT: 2023‐507839‐38‐00). Healthy women of non‐childbearing potential (permanently sterile or postmenopausal), 18‐65 years of age and with no laboratory abnormalities were eligible to participate. Full inclusion and exclusion criteria are listed in the Supporting Information. A daily dose of ganfeborole 20 mg was selected for the study, based on the efficacy data of a Phase 2a early bactericidal activity study.^12^

**Figure 2:**
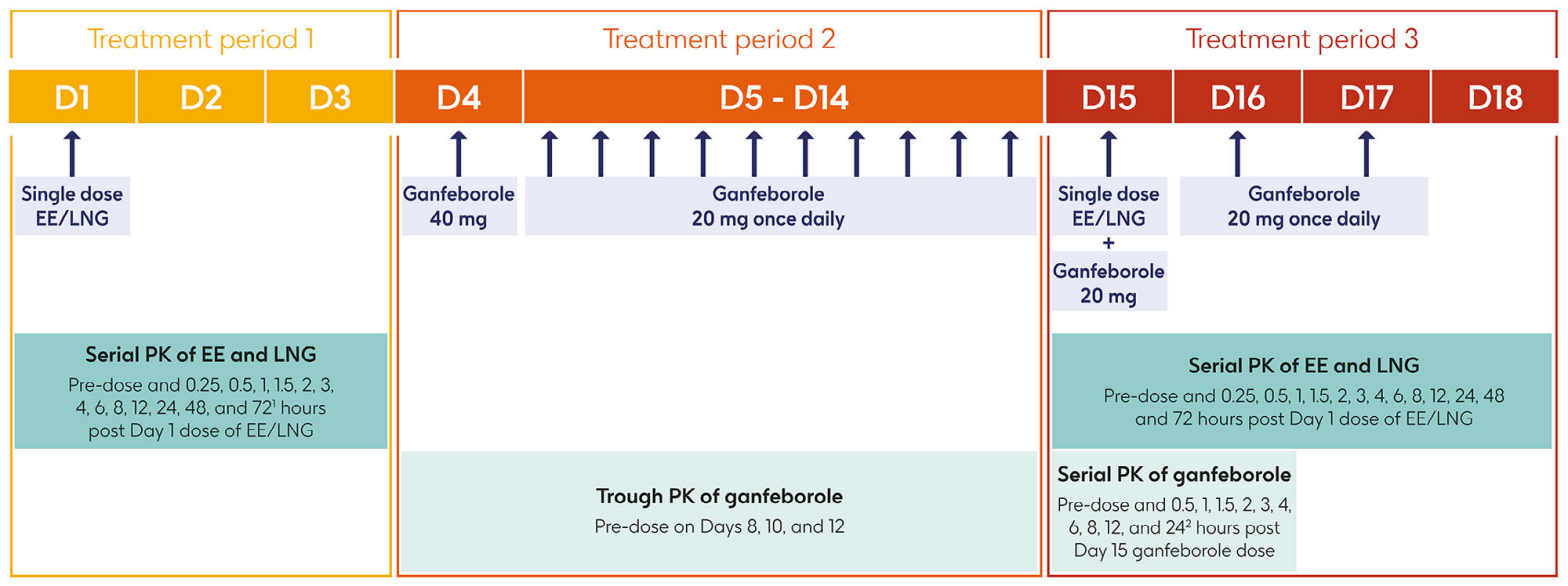
Study design. ^1^72‐hour sample taken before ganfeborole dose on Day 4; ^2^24‐hour sample taken before ganfeborole administration on Day 16. D, day; EE, ethinyl estradiol; LNG, levonorgestrel; PK, pharmacokinetics.

Screening took place during the 14 days prior to the first study treatment, and the study consisted of three treatment periods (Figure 2). During treatment period 1, participants entered the study clinic on the evening before Day 1 and received a single dose of EE/LNG on Day 1. Participants took EE/LNG in the morning following an overnight fast of at least 8 h and remained fasted for at least 4 h. During treatment period 2, participants received a loading dose of ganfeborole 40 mg (GSK) on Day 4 to ensure appropriate steady‐state conditions were achieved by the time of EE/LNG co‐administration. Participants were then discharged from the clinic, and self‐administered maintenance doses of ganfeborole 20 mg once daily from Days 5 to 14 at home, at the same time each day. For treatment period 3, participants re‐entered the clinic on the evening before Day 15 and received a single dose of EE/LNG co‐administered with ganfeborole 20 mg on Day 15, followed by ganfeborole 20 mg once daily on Days 16 and 17. Participants were discharged on Day 18. As food is not expected to impact on ganfeborole exposure based on Phase 1 data,^10^ there were no dietary restrictions or fasting requirements for ganfeborole administration in this study.

Study medication was taken at the clinic under medical supervision and recorded in the study documentation. Adherence to study medication taken at home was assessed at each visit by direct questioning of participants and counting returned capsules, and recorded in the study documentation. In addition, participants recorded medication adherence in a diary.

Blood samples for serial PK analysis of EE and LNG were collected pre‐dose and 0.25, 0.5, 1, 1.5, 2, 3, 4, 6, 8, 12, 24, 48, and 72 h after the Days 1 and 15 dose of EE/LNG (Figure 2). The 72‐h sample was taken before administration of ganfeborole on Day 4. For the trough PK analysis of ganfeborole, blood samples were collected 24 h after the previous ganfeborole dose and within 30 min before the next dose on Days 8, 10, and 12 (Figure 2). Blood samples for the serial PK analysis of ganfeborole were collected pre‐dose and 0.5, 1, 1.5, 2, 3, 4, 6, 8, 12, and 24 h after the Day 15 dose of ganfeborole (Figure 2). The pre‐dose sample was taken within 30 min before ganfeborole administration, and the 24‐h sample was taken before the Day 16 dose.

Analytical methods were developed by Labcorp (Madison, USA), who also conducted the laboratory analysis for this study. The concentrations of EE and LNG were measured using methodology proprietary to Labcorp in human plasma specimens containing K2 ethylenediamine tetra acetic acid (EDTA) as an anticoagulant, using supported‐liquid extraction and derivatization followed by high performance liquid chromatography (HPLC) with tandem mass spectrometry (MS/MS) detection using ethinyl estradiol‐d_4_ (stable‐label) and norgestrel‐d_6_ (stable‐label) as internal standards for EE and LNG, respectively. The method was validated by Labcorp. For EE, the validated range was 7.50‐500 pg/mL and recovery was in the range of 88.7%‐103.2%. For LNG, the validated range was 150‐10,000 pg/mL and recovery was in the range of 90.4%‐98.4%. Ganfeborole concentration was measured in human plasma specimens containing K2 EDTA as an anticoagulant, using solid phase extraction followed by HPLC on a Waters Acquity BEH C18 column with MS/MS detection using [^11^B, ^13^C] GSK2982434‐d_6_ (stable‐label) as an internal standard. An electrospray ion source was used for ionization and measurements were done in the positive ionization mode. The method was validated by Labcorp. The validated range was 2.00‐2000 ng/mL and recovery was in the range of 60.3%‐63.8%.

### Pharmacokinetic Objectives and Endpoints

The primary objective was to assess the effect of ganfeborole steady‐state exposure on the single dose PK of EE and LNG, measured by comparing EE and LNG PK parameters after EE/LNG alone (treatment period 1) versus after concomitant EE/LNG plus ganfeborole (treatment period 3). The endpoints assessed were the area under the plasma concentration‐time curve extrapolated to infinity (AUC_(0‐inf)_) and maximum concentration (*C*_max_) of EE and LNG.

A secondary objective was to assess the steady‐state PK of ganfeborole in the presence of EE and LNG. PK parameters determined were AUC_(0‐τ)_ (where τ represents the end of the dosing interval), *C*_max_, time to *C*_max_ (*t*_max_), and *C_τ_* on Days 8, 10, 12, 15, and 16. Another secondary objective was to further assess the single dose PK of EE and LNG alone (treatment period 1) and in the presence of ganfeborole (treatment period 3), based on AUC from 0 to the time of last quantifiable concentration (AUC_(0‐t)_), *t*_max_, and half‐life (*t*_½_).

### Safety Assessments

Adverse events (AEs), serious adverse events (SAEs), and laboratory values were recorded. AEs were reported according to the Common Terminology Criteria for Adverse Events (CTCAE).

### Statistical Analysis

Safety was analyzed in participants who received at least one dose of study medication (EE/LNG or ganfeborole). PK analysis was performed in participants who received at least one dose of study medication and had at least one non‐missing PK assessment. In addition, to be included in the analysis of the primary endpoint, participants had to have missed no more than one ganfeborole dose during treatment period 2 and to have been 100% compliant with study medication during treatment periods 1 and 3.

PK data were analyzed using non‐compartmental methods with Phoenix WinNonlin Version 8.3 to derive PK parameters. EE and LNG AUC_(0‐inf)_ and *t*_½_ were flagged but included in the PK analysis if: (i) the percentage of AUC_(0‐inf)_ obtained by extrapolation was >20% but ≤40%, and/or (ii) the interval used to determine the apparent terminal phase rate constant (*λ*_z_) via regression of the PK data in loglinear scale was less than twice the t_½_. EE and LNG AUC_(0‐inf)_ and t_½_ were excluded from the PK analysis if: (i) the percentage of AUC_(0‐inf)_ obtained by extrapolation (%AUC_ex_) was >40%, and/or (ii) the adjusted coefficient of determination (*R* ^2^_adj_) of the *λ*_z_ regression was <0.85.

Descriptive statistics were calculated for the primary and secondary endpoints. For the primary endpoint, log_e_‐transformed PK parameters were analyzed separately using linear mixed effect models with treatment as a fixed effect and participant as a random effect. Point estimates for the ratio of the geometric means (EE or LNG plus ganfeborole versus EE or LNG alone) and 90% confidence intervals (CI) for AUC_(0‐inf)_ and *C*_max_ were calculated. If the 90% CI of the geometric mean ratios were within a range of 0.80‐1.25, for EE and LNG AUC_(0‐inf)_ and *C*_max_, a lack of a drug–drug interaction could have been concluded.

To assess the steady‐state PK of ganfeborole, the natural logarithm of *C_τ_* was analyzed using a mixed effect model with day (Days 8, 10, 12, and 15 pre‐dose concentration) as a fixed effect and participant as a random effect. The coefficients of the slopes for the day effect on a log scale, along with corresponding 90% CIs, were used to determine whether ganfeborole steady‐state was achieved prior to co‐administration with EE/LNG.

The sample size was based on the within‐participant coefficient of variation (CVw, %) calculated from previously reported^19^ adjusted geometric mean ratios and 90% CI using the CVfromCI function of the POWERTOST statistical package in R software. The ratio of exposure for EE or LNG plus ganfeborole versus EE or LNG alone was assumed to be 1. To calculate the joint power of the four comparisons (AUC_(0‐inf)_ and *C*_max_ for EE and LNG), a conservative between‐parameter correlation of 0.6 was assumed. Based on these assumptions, a sample size of 16 provided 92% joint power to ensure that the 90% CIs for the geometric mean ratios for all parameters lay within the range of 0.80‐1.25. To account for an expected 20% rate of non‐evaluable participants, approximately 20 participants were planned to be enrolled.

## Results

The study took place between 5 April and July 1, 2024. Database lock was on the 18th of September 2024. A total of 28 participants were screened, 20 were enrolled, and 18 completed the study (Figure S1). All enrolled participants were included in the safety analysis and 19 were included in the PK analysis, of whom one participant had EE and LNG PK data available for treatment period 1 only. All 18 participants who completed the study were fully compliant with medication.

All participants were White. The mean age was 53.6 years (standard deviation 5.6), and median body mass index was 24.9 kg/m^2^ (range 20.7 to 30.8). Three participants of childbearing age were sterile and 17 participants were postmenopausal.

### Pharmacokinetics of EE and LNG

Plasma concentrations of EE were quantifiable up to at least 24 h post‐dose in 14/19 participants in treatment period 1 and up to at least 12 h post‐dose in 16/18 participants in treatment period 3. Plasma concentrations of LNG were quantifiable up to 72 h post‐dose in 9/19 participants in treatment period 1 and 11/18 participants in treatment period 3.

**Figure 3:**
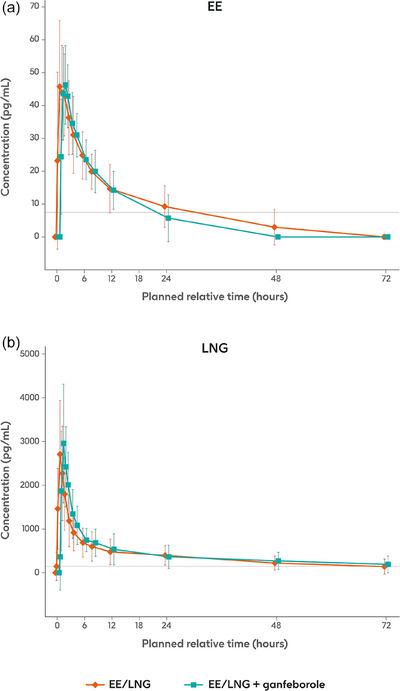
Mean (SD) jittered plasma concentration‐time profiles of EE and LNG after single‐dose administration of EE/LNG alone (Day 1) or co‐administered with ganfeborole (Day 15). Horizontal lines are the lower limits of quantification: 7.5 pg/mL for EE and 150 pg/mL for LNG. EE, ethinyl estradiol; LNG, levonorgestrel; SD, standard deviation.

**Figure 4:**
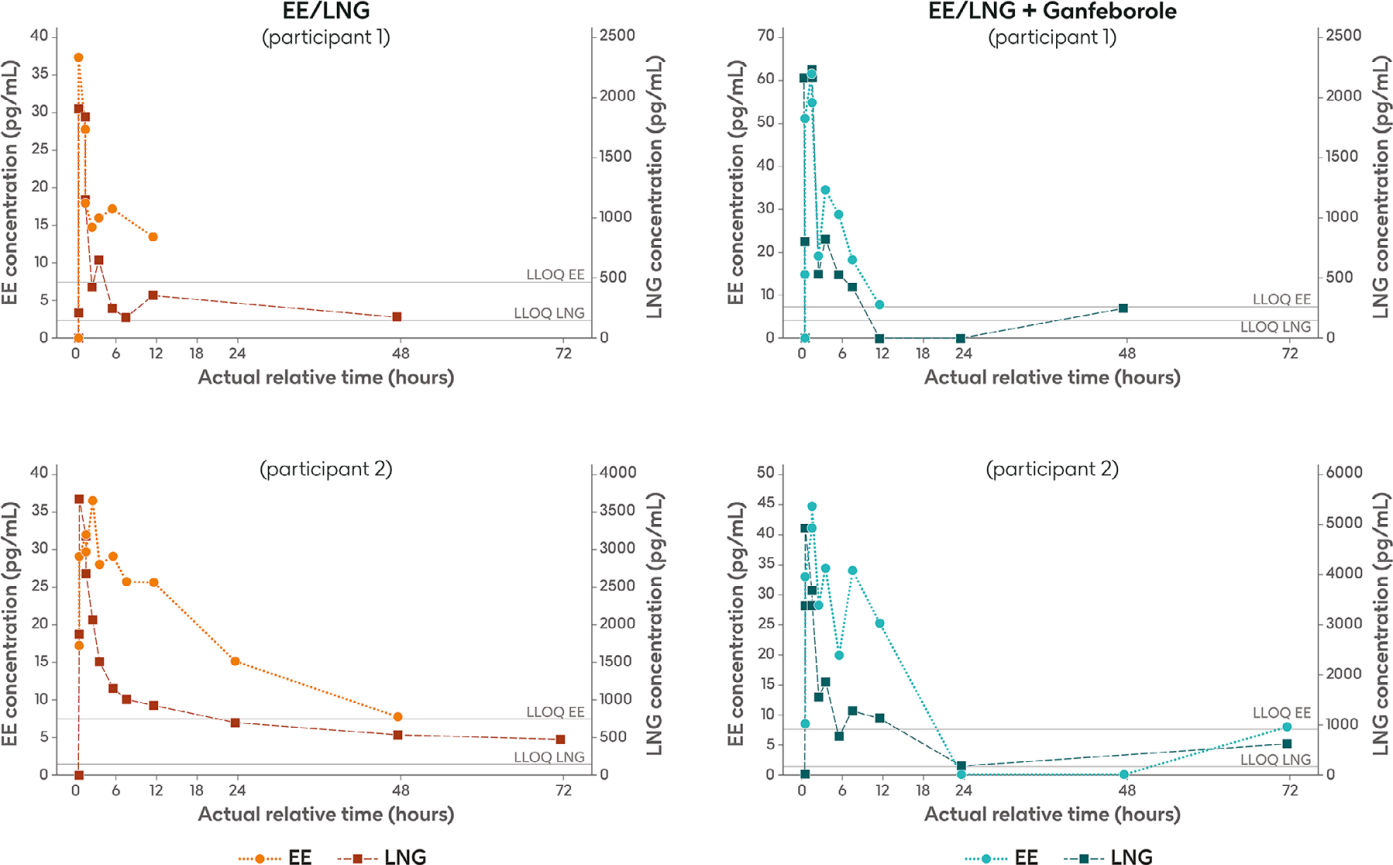
Individual plasma concentration‐time profiles of EE and LNG for two participants after single‐dose administration of EE/LNG alone (Day 1) or co‐administered with ganfeborole (Day 15). Horizontal lines are the lower limits of quantification: 7.5 pg/mL for EE and 150 pg/mL for LNG. EE, ethinyl estradiol; LLOQ: lower limit of quantification; LNG, levonorgestrel.

The summary statistics of the PK parameters of EE and LNG are shown in Table 1. The mean EE and LNG concentration‐time profiles after coadministration of EE/LNG plus ganfeborole on Day 15 were broadly comparable to those observed after administration of EE/LNG alone on Day 1 (Figure 3). Notably, numerous individual plasma concentration‐time profiles for both EE and LNG presented unexpected fluctuations around *C*_max_ and in the terminal phase in both treatment periods (Figure 4). This implied that estimates of AUC_(0‐inf)_ for EE did not meet acceptability criteria (*R* ^2^_adj_ ≥0.85 and/or %AUC_ex_ ≤40%) in 13/19 participants during treatment period 1 and 10/18 participants during treatment period 3. Similarly, estimates of AUC_(0‐inf)_ for LNG did not meet acceptability criteria in 11/19 participants during treatment period 1 and 9/18 participants during treatment period 3. AUC_(0‐inf)_ values not meeting acceptability criteria were therefore not included in the summary statistics and statistical analysis.

**Table 1:** Pharmacokinetics of EE and LNG After Single‐Dose Administration of EE/LNG Alone (Day 1) or Co‐Administered With Ganfeborole (Day 15)

	EE		LNG	
	EE/LNG alone N = 19	EE/LNG + ganfeborole N = 18	EE/LNG alone N = 19	EE/LNG + ganfeborole N = 18
***C*_max_ (pg/mL), n**	19	18	19	18
Geometric mean (%CVb)	50.86 (32.7)	48.96 (24.5)	2599.85 (46.8)	2828.17 (45.3)
***t*_max_ (h), n**	19	18	19	18
Median (range)	0.967 (0.47, 2.97)	1.433 (0.45, 3.05)	0.967 (0.92, 1.93)	0.967 (0.45, 2.07)
**AUC_(0‐_ *_t_*_)_ (h·pg/mL), n**	19	18	19	18
Geometric mean (%CVb)	447.10 (58.7)	363.00 (43.4)	21,182.61 (84.3)	23,287.77 (91.3)
**AUC_(0‐inf)_ (h·pg/mL), n**	6	8	8	9
Geometric mean (%CVb)	627.11 (53.0)	552.01 (51.0)	24,651.76 (113.2)	30,334.48 (94.2)
***t*_½_ (h), n**	13	10	12	13
Median (range)	19.180 (4.38, 49.03)	10.867 (3.14, 29.39)	41.315 (4.00, 100.00)	34.697 (4.17, 118.18)

AUC_(0‐_ *_t_*_)_, area under the concentration time curve from time 0 to the time of the last quantifiable concentration; AUC_(0‐inf)_, area under the concentration time curve from time 0 to infinity; *C*_max_, maximum concentration; EE, ethinyl estradiol; LNG, levonorgestrel; N, number of participants per treatment period; n, number of participants with acceptable pharmacokinetic parameter value; *t*_½_, half‐life; *t*_max_, time to maximum concentration; %CVb, between‐participant coefficient of variation.

The geometric mean ratio of EE AUC_(0‐inf)_ (EE/LNG + ganfeborole versus EE/LNG) was 0.88 (90% CI: 0.55, 1.41) (Table 2). As the 90% CIs did not fall within the pre‐defined range of 0.80‐1.25, lack of interaction between EE and ganfeborole could not be concluded based on this parameter. However, the geometric mean ratio of EE *C*_max_ was 0.96 (90% CI: 0.85, 1.09), the geometric mean ratio of LNG AUC_(0‐inf)_ was 1.10 (90% CI: 0.98, 1.23), and the geometric mean ratio of LNG *C*_max_ was 1.08 (90% CI: 0.97, 1.19) (Table 2), thus all meeting the pre‐defined criterion for lack of a drug–drug interaction.

**Table 2:** Geometric Mean Ratios of AUC_(0–inf)_ and *C*_max_ for EE and LNG Comparing EE/LNG Co‐Administered With Ganfeborole Versus EE/LNG Administered Alone

Parameter	Study drug	N	n	Adjusted geometric mean (90% CI)	Ratio (90% CI)^a^	CV, %^a^
**EE**						
AUC_(0‐inf)_ (h·pg/mL), n	EE/LNG	19	6	627.11 (439.7, 894.4)	0.88 (0.55, 1.41)	51.85
	EE/LNG + ganfeborole	18	8	552.01 (405.9, 750.7)		
*C*_max_ (pg/mL), n	EE/LNG	19	18	50.86 (45.3, 57.0)	0.96 (0.85, 1.09)	21.59
	EE/LNG + ganfeborole	18	18	48.96 (43.6, 54.9)		
**LNG**						
AUC_(0‐inf)_ (h·pg/mL), n	EE/LNG	19	8	25,578.6 (16,767.5, 39,019.9)	1.10 (0.98, 1.23)	8.53
	EE/LNG + ganfeborole	18	9	28,063.0 (18,407.4, 42,783.5)		
*C*_max_ (pg/mL), n	EE/LNG	19	18	2624.1 (2190.3, 3143.8)	1.08 (0.97, 1.19)	17.6
	EE/LNG + ganfeborole	18	18	2828.2 (2360.6, 3388.3)		

^a^ EE/LNG + ganfeborole versus EE/LNG alone.

AUC_(0‐inf)_, area under the concentration time curve from time 0 to infinity; CI, confidence interval; *C*_max_, maximum concentration; CV, coefficient of variation; EE, ethinyl estradiol; LNG, levonorgestrel; N, number of participants per treatment period; n, number of participants with acceptable pharmacokinetic parameter value.

### Post‐hoc Analysis

Because of the high number of non‐acceptable AUC_(0‐inf)_ values, post‐hoc analyses were conducted to provide additional insights. EE and LNG AUC_(0‐8)_ and AUC_(0‐24)_ were calculated and, following log‐transformation, were statistically analyzed using the same approach as the primary analysis. These partial AUCs were selected because 8 h was the last timepoint for which all participants had quantifiable concentrations of EE and LNG, and 24 h corresponds to the therapeutic dosing interval for EE/LNG.

The geometric mean ratio for EE AUC_(0‐8)_ (EE/LNG + ganfeborole versus EE/LNG) was 1.00 (90% CI: 0.90, 1.12) and the geometric mean ratio for AUC_(0‐24)_ was 0.97 (90% CI: 0.86, 1.09) (Table 3). The geometric mean ratio for LNG AUC_(0‐8)_ was 1.14 (90% CI: 1.06, 1.23) and the geometric mean ratio for LNG AUC_(0‐24)_ was 1.11 (90% CI: 1.02, 1.20) (Table 3). The results of these four post‐hoc analyses indicate a lack of drug‐drug interaction according to the criterion of being within the 0.80‐1.25 range (Table 3).

**Table 3:** Geometric Mean Ratios of AUC_(0‐8)_ and AUC_(0‐24)_ for EE and LNG Comparing EE/LNG Co‐Administered with Ganfeborole Versus EE/LNG Administered Alone (Post‐hoc Analysis)

Parameter	Study drug	N	n	Adjusted geometric mean (90% CI)	Ratio (90% CI)^b^	CV, %^b^
**EE**						
AUC_(0‐8)_ (h·pg/mL),^a^ n	EE/LNG	19	18	229.1 (207.0, 253.6)	1.00 (0.90, 1.12)	19.52
	EE/LNG + ganfeborole	18	18	229.4 (207.3, 253.9)		
AUC_(0‐24)_ (h·pg/mL),^a^ n	EE/LNG	19	17	448.8 (401.1, 502.2)	0.97 (0.86, 1.09)	17.15
	EE/LNG + ganfeborole	18	13	434.5 (384.0, 491.6)		
**LNG**						
AUC_(0‐8)_ (h·pg/mL),^a^ n	EE/LNG	19	18	8120.8 (6789.8, 9712.5)	1.14 (1.06, 1.23)	13.10
	EE/LNG + ganfeborole	18	18	9296.4 (7772.8, 11,118.6)		
AUC_(0‐24)_ (h·pg/mL),^a^ n	EE/LNG	19	18	14,459.8 (11,731.5, 17,822.6)	1.11 (1.02, 1.20)	14.07
	EE/LNG + ganfeborole	18	18	16,033.7 (13,008.4, 19,762.5)		

^a^ AUC_(0‐8)_ and AUC_(0‐24)_ were calculated via either interpolation (if time of last quantifiable concentration was >8 or >24 h, respectively) or via extrapolation (otherwise). If extrapolation was not feasible due to non‐acceptable apparent terminal phase rate constant (*λ*_z_), AUC_(0‐8)_ or AUC_(0‐24)_ were either imputed with AUC_(0‐_ *_t_*_)_ (if time of last quantifiable concentration, t, was within allowance window for the 8‐ or 24‐h timepoint, respectively) or not calculated (as non‐acceptable).

^b^ EE/LNG + ganfeborole versus EE/LNG alone.

AUC_(0‐8)_, area under the concentration time curve from time 0 to 8 h; AUC_(0‐24)_, area under the concentration time curve from time 0 to 24 h; CI, confidence interval; CV, coefficient of variation; EE, ethinyl estradiol; LNG, levonorgestrel; N, number of participants per treatment period; n, number of participants with acceptable pharmacokinetic parameter value.

### Pharmacokinetics of Ganfeborole

The mean concentration‐time profile of ganfeborole is shown in Figure S2. The summary statistics of ganfeborole PK parameters are reported in Table 4. Ganfeborole steady‐state was achieved by Day 8, i.e. at least 1 week before EE/LNG administration on Day 15 (Table S1).

**Table 4:** Pharmacokinetic Parameters of Ganfeborole Following a 40 mg Loading Dose (Day 4) and 20 mg Maintenance Doses (Days 5‐17)

Parameter	Reference dosing day	Treatment	n	Geometric mean (%CVb)*
*C_τ_* (ng/mL)	8	Ganfeborole alone	19	226.92 (17.9)
*C_τ_* (ng/mL)	10	Ganfeborole alone	19	235.54 (27.5)
*C_τ_* (ng/mL)	12	Ganfeborole alone	19	243.40 (21.9)
*C_τ_* (ng/mL)	15	Ganfeborole + EE/LNG	18	251.26 (40.3)
*C_τ_* (ng/mL)	16	Ganfeborole + EE/LNG	18	254.91 (23.0)
*C*_max_ (ng/mL)	15	Ganfeborole + EE/LNG	18	600.62 (14.2)
*t*_max_ (h)	15	Ganfeborole + EE/LNG	18	0.95 (0.4, 3.0)
AUC_(0‐_ *_τ_*_)_ (h·ng/mL)	15	Ganfeborole + EE/LNG	18	8372.42 (18.8)

AUC_(0‐_ *_τ_*_)_, area under the concentration time curve over the dosing interval (24 h); *C*_max_, maximum concentration; CVb, between‐participant coefficient of variation; *C_τ_*, pre‐dose (trough) concentration; *t*_max_, time to maximum concentration.

* Data are geometric mean (%CVb) except *t*_max_ which is median (range).

### Safety

Six participants reported six AEs. In treatment period 1 (EE/LNG alone), 4/20 (20%) participants reported an AE. In treatment period 2 (ganfeborole alone), 1/19 (5%) participants reported an AE. In treatment period 3 (EE/LNG plus ganfeborole), 1/18 (6%) participants reported an AE. All were Grade 1 (mild) in the CTCAE category. Headache was the most common AE, reported by four participants in the EE/LNG alone treatment period and one participant in the EE/LNG plus ganfeborole treatment period. One participant discontinued the study during treatment period 2 after experiencing a dental abscess that required antibiotics and an extraction. No AEs were considered related to study medication and none led to a dose reduction, interruption, or delay. No SAEs or deaths were reported. No clinically meaningful changes in laboratory parameters were observed.

## Discussion

Several potential combination regimens for the treatment of TB carry label restrictions for women who are pregnant and women of childbearing potential, limiting treatment options for this population. The ability to co‐administer ganfeborole with oral contraceptives without compromising their efficacy will allow broader access to ganfeborole among women of childbearing potential.

This study was conducted in accordance with the European Medicines Agency scientific guideline on the investigation of drug–drug interactions.^15^ The guideline notes that a drug with potential effects on embryofetal development needs to be studied in vivo for effects on contraceptive steroids if the drug is intended for use in fertile women, and in vitro data alone should not be relied upon to predict lack of any potentially clinically significant interaction.^15^ United States Food and Drug Administration guidance also indicates that a drug–drug interaction study is recommended for licensure of a potential human teratogen.^16^

The study showed unexpected fluctuations in individual EE and LNG plasma concentration‐time profiles, irrespective of whether EE/LNG was dosed alone or with ganfeborole. As a result, more than half of EE and LNG AUC_(0‐inf)_ estimates from evaluable participants did not meet acceptability criteria, leading to exclusions from the primary statistical analysis. We investigated several potential reasons for the unexpected fluctuations in EE and LNG [data on file; GSK document No. TMF‐20496559]. To verify the numerical PK results, we explored a potential swap in timepoints in the dataset used for PK parameter derivation, any inconsistency between values in the dataset and the PK file from Labcorp that had undergone quality control checking, the possibility of bioanalytical issues, expiry date of the EE/LNG product, or issues with collection or processing of the PK samples at the study site. However, no issues were found for any of these points.

Additionally, we considered whether the fluctuations could be explained by other demographic‐ or design‐related factors: menopausal status of the participant (post‐menopausal versus pre‐menopausal with a tubal ligation); higher body mass index (BMI), potentially leading to higher endogenous estrogens that confounded the bioanalytical assay; variations in sex hormone binding globulin (SHBG), leading to non‐linear binding to EE; enterohepatic recirculation; and food conditions. However, all these potential root causes were discounted. Indeed, only one participant was pre‐menopausal, and, in general, the PK of EE and LNG are not expected to differ between pre‐ and post‐menopausal women.^20^ Participants with either normal or high BMI displayed erratic PK profiles. No correlation between SHBG values at screening and observed PK fluctuations was observed, and post‐menopausal women are not expected to have cyclical fluctuations in SHBG.^21^ Enterohepatic recirculation could have caused second or multiple peaks of EE, but this could not explain the erratic fluctuations observed for both EE and LNG. Food conditions were also not deemed to be the root cause, as all participants had a similar fasting time and time from administration to the first meal, and times of PK fluctuation were not related to time of meal intake.

Ganfeborole PK was consistent with previous studies, as *C*_max_ and AUC_(0‐_ *_τ_*_)_ were on average within the exposure previously observed for repeat daily doses of 15 and 30 mg.^10^, ^12^

The primary statistical analysis indicated no interaction between ganfeborole and LNG, as the 90% CIs for the ratio of the geometric means for EE/LNG plus ganfeborole versus EE/LNG alone for LNG AUC_(0‐inf)_ and *C*_max_ were within the range of 0.80‐1.25. The same was observed for the 90% CI associated with the *C*_max_ for EE. However, the 90% CI associated with EE AUC_(0‐inf)_ was wider than 0.80‐1.25. Therefore, a lack of drug–drug interaction between ganfeborole and EE/LNG could not be concluded based on the primary analysis. Considering the challenges in derivation of the primary endpoints (EE and LNG AUC_(0‐inf)_), post‐hoc analyses based on partial AUCs, up to 8 and 24 h post‐dose, were conducted. The use of partial AUCs allowed for a more reliable PK comparison between treatment periods by reducing the potential bias introduced by extrapolation procedures that affected the primary endpoint calculations. These post‐hoc analyses supported a lack of drug–drug interaction between ganfeborole and EE/LNG, as the 90% CIs for the ratio of the geometric means for LNG AUC_(0‐8)_, LNG AUC_(0‐24)_, EE AUC_(0‐8)_, and EE AUC_(0‐24)_ were all within the 0.80‐1.25 range. Whilst a drug–drug interaction cannot be ruled out based on the present study, results were confounded by the unexpected fluctuations in EE and LNG PK profiles, resulting in a much smaller sample size for analysis of the primary endpoints, particularly for EE AUC_(0‐inf)_ (n = 6 and 8 for EE/LNG and EE/LNG + ganfeborole respectively; the planned sample size was 16 evaluable participants per group).

## Conclusions

The unexpected fluctuations in the PK profiles of EE and LNG observed led to a limited number of acceptable primary endpoints, resulting in CIs that were outside the range specified for no drug interaction. The data are therefore inconclusive regarding the absence of a drug–drug interaction. To generate definitive data, a repeat study further exploring potential drug–drug interactions between ganfeborole and combined oral contraceptives would be required. The current strategy recommended for future trials is to continue the use of highly effective, non‐user dependent contraceptive methods.

## Author Contributions

LI, SML, TC, RG‐C, SG, SLP, KR, ST, DB‐A, and AMB contributed to the study concept or design. AGLH, AM‐C, and AMB were responsible for data acquisition. LI, SML, AJC, TC, RG‐C, SG, AM‐C, SLP, KR, ST, DB‐A, and AMB performed data analysis, and/or data interpretation. All authors contributed to the development of the manuscript by reviewing and providing input. All authors had full access to the data and gave final approval before submission. All authors agree to be accountable for all aspects of the work.

## Conflicts of Interest

LI, and SML provided consultancy to GSK. TC, RG‐C, SG, SLP, KR, ST, and DB‐A are GSK employees. RG‐C, SG, SLP, KR, ST, and DB‐A hold financial equities in GSK. SLP, KR, and DB‐A hold financial equities in Haleon. DB‐A also reports patents planned, issued or pending and the following grants paid to his institution: ERA4TB (CDTI) (IDI‐20200356); bETO‐TB supported by the European Union (RIA2019AMR‐2657); CLICK‐TB ‐ supported by the European Union (RIA2017T‐2030). AJC reports the following paid to his institution: industry‐funded professional development programme from the Universidad Autónoma de Madrid and AbbVie; investigation grants from European and national funding agencies; and contracts related to research and principal investigator roles in clinical trials. AJC also reports payment for scientific reviews of public investigation calls (ISCIII and others). AGLH received a grant from the Fundación para la Investigación Biomédica Hospital Universitario La Paz within the past 36 months. AM‐C declares no conflicts of interest. All authors declare no other financial and non‐financial relationships and activities.

## Funding

The project leading to this publication has received funding from the Innovative Medicines Initiative 2 Joint Undertaking (JU) under grant agreement No. 853989. The JU receives support from the European Union's Horizon 2020 Research and Innovation Programme and EFPIA and Global Alliance for TB Drug Development Non‐Profit Organization, the Bill & Melinda Gates Foundation, and the University of Dundee. This work reflects only the author's views, and the JU is not responsible for any use that may be made of the information it contains. GSK is the sponsor of the study (NCT06354257; EudraCT: 2023‐507839‐38‐00) and was involved in all stages of research conduct, including analysis of the data. GSK also took in charge all costs associated with the development and publication of this manuscript.

## Supporting information



SUPPORTING INFORMATION

## Data Availability

Anonymized individual participant data and study documents can be requested for further research from www.clinicalstudydatarequest.com.
