# Supplementary material for: Combined Oral Contraceptive Drug–Drug Interaction Study With Ganfeborole, a New Anti‐Tuberculosis Agent
Source: J Clin Pharmacol. 2026 Mar 6;66(3):e70161. doi: 10.1002/jcph.70161 (PMC12966810; doi:10.1002/jcph.70161)
Supplement: Supplementary file 1 — SUPPORTING INFORMATION [file JCPH-66-0-s001.docx]

Supplementary Material

**Manuscript Title:** Combined oral contraceptive drug-drug interaction study with ganfeborole, a new anti-tuberculosis agent

**Authors:** Laura Iavarone, Silvia M. Lavezzi, Antonio J. Carcas, Tetyana Chaychenko, Raquel Gabarro-Carrion, Arturo Gómez López de las Huertas, Stephanie Gresham, Alicia Marín-Candón, Sophie L. Penman, Katie Rolfe, Simon Tiberi, David Barros-Aguirre, Alberto M. Borobia, on behalf of the ERA4TB consortium

## Inclusion criteria

1. Aged 18 to 65 years, inclusive, at the time of signing the informed consent form.

2. Healthy or compensated as determined by the investigator or medically qualified designee based on a medical evaluation, including medical history, physical examination, laboratory tests and cardiac monitoring (history and electrocardiogram).

3. Creatinine clearance ≥ 75 mL/min (Cockroft–Gault formula).

4. Normal echocardiogram or echocardiogram with normal left ventricular function with at most trace to mild valvular regurgitation is allowed and no valvular stenosis.

5. Body weight ≥ 45.0 kg and body mass index within the range 18.5 to 31.0 kg/m^2^ (inclusive).

6. Female of nonchildbearing potential, defined as:

a. Permanently sterile due to one of the following procedures: documented hysterectomy, bilateral salpingectomy or bilateral oophorectomy

- For permanently sterile individuals due to an alternate medical cause other than the above, (e.g. Mullerian agenesis, androgen insensitivity or gonadal dysgenesis), investigator discretion should be applied to determine study entry. If reproductive status is questionable, additional evaluation should be considered.

b. Postmenopausal female, defined as no menses for 12 months without an alternative medical cause.

- A high level of follicle-stimulating hormone in the postmenopausal range may be used to confirm a postmenopausal state in women not using hormonal contraception or hormone replacement therapy (HRT). However, in the absence of 12 months of amenorrhoea, confirmation with more than one measurement of follicle-stimulating hormone is required, within the screening period.
- Females on HRT and whose menopausal status is in doubt must discontinue HRT at least 30 days prior to the start of treatment period 1, to allow confirmation of postmenopausal status before study enrolment.

7. Participant must be capable of giving signed informed consent, which includes compliance with the requirements and restrictions listed in the informed consent form and in the protocol.

## Exclusion criteria

1. History of known cardiac valve abnormalities.

### Laboratory assessments

2. Presence of hepatitis B surface antigen at screening or within 3 months prior to starting study treatment.

3. Positive hepatitis C antibody test result at screening or within 3 months prior to starting study treatment AND positive on reflex to hepatitis C RNA.

4. Positive human immunodeficiency virus (HIV)-1 and -2 antigen/antibody immunoassay at screening.

5. Alanine aminotransferase (ALT) > 1.5 × upper limit of normal (ULN). A single repeat of ALT is allowed within a single screening period to determine eligibility.

6. Bilirubin > 1.5 × ULN (isolated bilirubin > 1.5 × ULN is acceptable if bilirubin was fractionated and direct bilirubin < 35%).

7. Any acute laboratory abnormality at screening which, in the opinion of the investigator, should preclude participation in the study of an investigational compound.

8. Participants with haemoglobin < 8.0 g/dL.

9. Any Grade 2 to 4 laboratory abnormality at screening, with the exception of creatine phosphokinase and lipid abnormalities (e.g. total cholesterol, triglycerides, etc.) and ALT (described above), excludes a participant from the study unless the investigator provides a compelling explanation for the laboratory result(s) and has the assent of the sponsor. A single repeat of any laboratory abnormality is allowed within a single screening period to determine eligibility.

10. A positive test result for drugs of abuse (including marijuana), alcohol, or cotinine (indicating active current smoking) at screening or before the first dose of study treatment.

### Prior or concomitant therapy

11. Unable to refrain from the use of prescription or non-prescription drugs including vitamins, herbal and dietary supplements (including St John’s wort) within 7 days (or 14 days if the drug is a potential enzyme inducer) or five half-lives (whichever is longer) prior to the first dose of study treatment and for the duration of the study. (Note: acetaminophen/paracetamol at doses of ≤ 2 g/day and hydrocortisone cream 1% are permitted for use at any time during the study. Levothyroxine and omeprazole may also be used during the study, providing the participant has been on a stable dose for at least one month prior to the start of treatment period 1 and they maintain the same dose throughout the study. The dose of ethinyl estradiol/levonorgestrel should be administered at least 1 hour after the dose of levothyroxine or omeprazole). Other concomitant medications may be permitted on a case-by-case basis at the discretion of the medical monitor and the GSK ganfeborole team.

12. Treatment with any vaccine within 30 days prior to receiving study treatment.

13. Unwillingness to abstain from excessive consumption of any food or drink containing caffeine, grapefruit or grapefruit juice, Seville oranges, blood oranges, or pomelos or their fruit juices within 7 days prior to the first dose of study treatment(s) until the end of the study.

14. The study excluded participants who had undergone in vitro fertilisation (IVF) or other assisted reproductive techniques within 9 months prior to screening, or were participating in such programmes at the time of screening, or who planned to undergo IVF or other assisted reproductive techniques during the following year.

### Prior or concurrent clinical study experience

15. Participation in another concurrent clinical study or prior clinical study (with the exception of imaging trials) prior to the first dosing day in the current study: 30 days, five half-lives plus 10 days, or twice the duration of the biological effect of the investigational product (whichever was longer).

16. Where participation in the study resulted in donation of blood or blood products in excess of 500 mL within 56 days.

### Diagnostic assessments

17. Any significant arrhythmia or electrocardiogram (ECG) finding (e.g. symptomatic bradycardia, non-sustained or sustained atrial arrhythmias, non-sustained or sustained ventricular tachycardia, second‑degree atrioventricular block Mobitz Type II, or third‑degree atrioventricular block) which, in the opinion of the investigator or GSK medical monitor, would interfere with the safety of the individual participant.

18. Exclusion criteria for screening ECG (a single repeat was allowed for eligibility determination):

| Heart rate^1^ | < 50 or > 100 beats per minute |
| --- | --- |
| QTcF interval^1^,^2^ (Fridericia’s formula) | >450 ms |

A heart rate from 100 to 110 beats per minute could be rechecked by ECG or vital signs within 30 minutes to verify eligibility. A QT interval corrected for heart rate using Fridericia’s formula (QTcF interval) between 450 and 460 milliseconds could be rechecked by ECG within 30 minutes to verify eligibility.

The corrected QT (QTc) is the QT interval corrected for heart rate according to Fridericia’s formula (QTcF). It is either machine-read or manually over-read. The specific formula used to determine eligibility and discontinuation for an individual participant in this study was Fridericia’s formula.

### Other exclusion criteria

19. Participants with vitiligo.

20. Participants with hypertension or type 2 diabetes that could not be controlled with diet and exercise alone.

21. History of regular alcohol consumption within 6 months of the study defined as an average weekly intake of > 14 units. One unit is equivalent to 8 g of alcohol: a half‑pint (approximately 240 mL) of beer, one glass (125 mL) of wine or one measure (25 mL) of spirits.

22. Unable to refrain from tobacco- or nicotine-containing products within 3 months prior to screening.

23. History of sensitivity to any of the study medications, or components thereof, or a history of drug or other allergy that, in the opinion of the investigator or medical monitor, contraindicates their participation.

Supplementary table 1. Analysis of log-transformed pharmacokinetic parameters of ganfeborole assessing steady state (Days 8, 10, 12, and Day 15 prior to EE/LNG administration)

| **Parameter** | **Back transformed slope (90% CI)** |
| --- | --- |
| C_τ_ (ng/mL) | 1.0013 (1.0000, 1.0026) |

The coefficients of the slopes for the day effect were used to determine whether ganfeborole steady state was achieved prior to co-administration with EE/LNG. The analysis was performed using linear mixed effect model with day as a fixed effect and participant as a random effect.

CI, confidence interval; C_τ_, pre-dose (trough) concentration; EE, ethinyl estradiol; LNG, levonorgestrel

Supplementary figure 1. Participant disposition


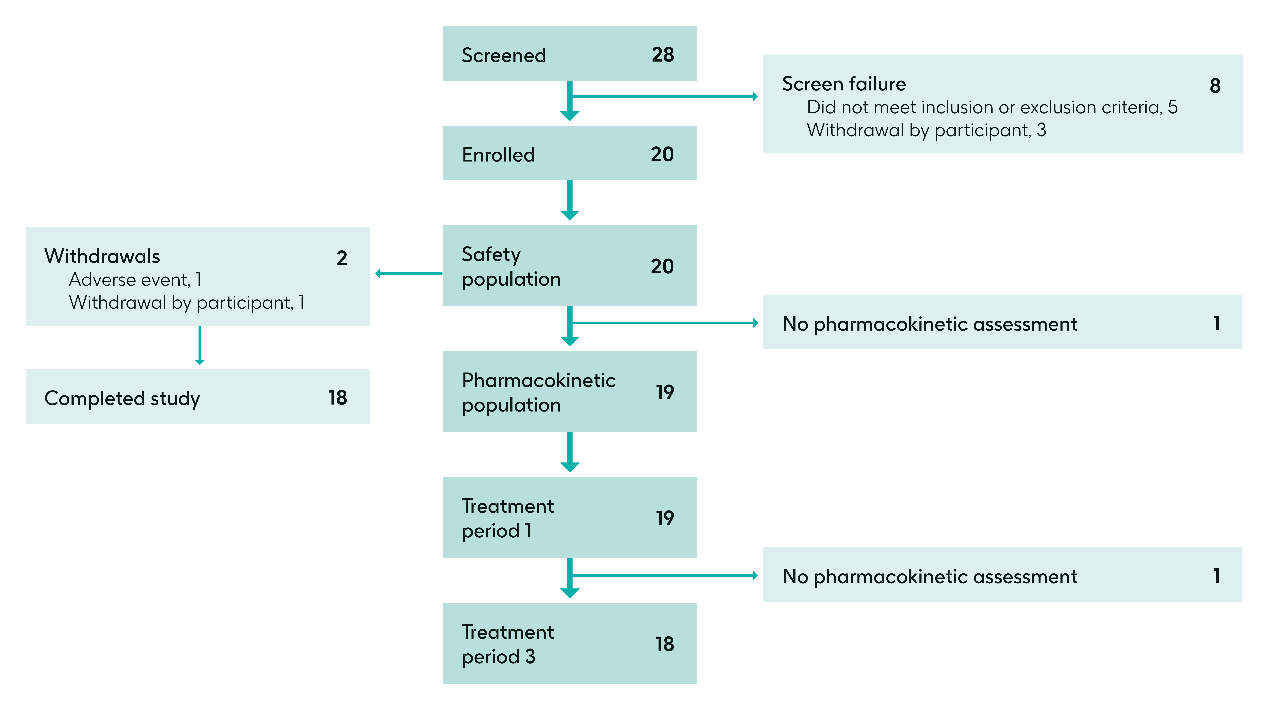


Supplementary figure 2. Mean (SD) plasma concentration-time profile of ganfeborole


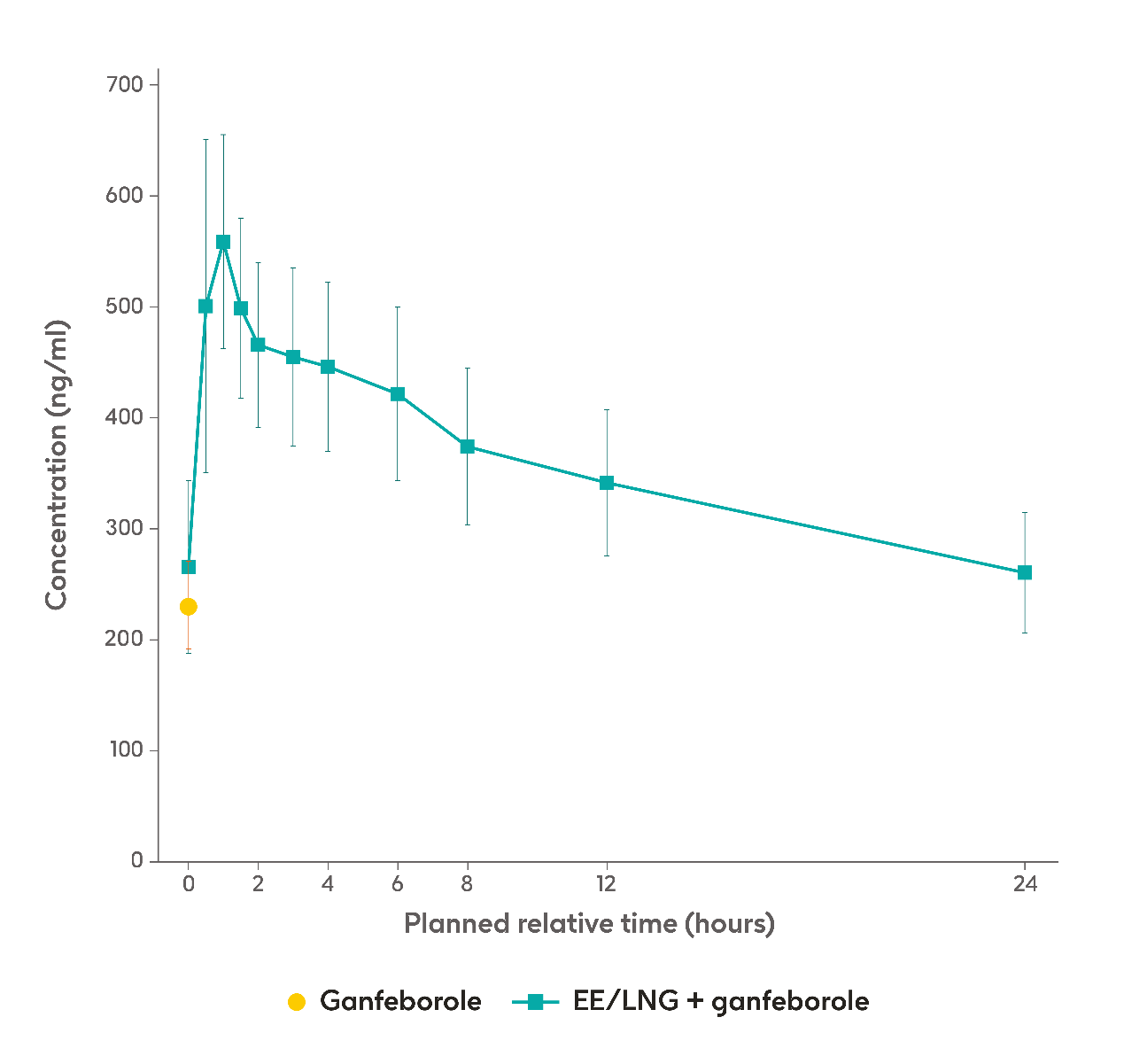


Lower limit of quantification for ganfeborole is 2 ng/mL.

EE: ethinyl estradiol; LNG: levonorgestrel; SD: standard deviation
